# Time Series Analyses of Hand, Foot and Mouth Disease Integrating Weather Variables

**DOI:** 10.1371/journal.pone.0117296

**Published:** 2015-03-02

**Authors:** Yuanbin Song, Fan Wang, Bin Wang, Shaohua Tao, Huiping Zhang, Sai Liu, Oscar Ramirez, Qiyi Zeng

**Affiliations:** 1 Pediatric Center of Zhujiang Hospital, Southern Medical University, Guangzhou, China; 2 Dept. of Biostatistics and Epidemiology, University of Pennsylvania, Philadelphia, PA, United States of America; 3 Yale Cancer Center, Yale University School of Medicine, New Haven, CT, United States of America; 4 Dept. of Psychiatry, Yale University School of Medicine, New Haven, CT, United States of America; 5 Dept. of Psychology, Yale University, New Haven, CT, United States of America; University of Washington, UNITED STATES

## Abstract

**Background:**

The past decade witnessed an increment in the incidence of hand foot mouth disease (HFMD) in the Pacific Asian region; specifically, in Guangzhou China. This emphasized the requirement of an early warning system designed to allow the medical community to better prepare for outbreaks and thus minimize the number of fatalities.

**Methods:**

Samples from 1,556 inpatients (hospitalized) and 11,004 outpatients (non-admitted) diagnosed with HFMD were collected in this study from January 2009 to October 2013. Seasonal Autoregressive Integrated Moving Average (SARIMA) model was applied to establish high predictive model for inpatients and outpatient as well as three viral serotypes (EV71, Pan-EV and CA16). To integrate climate variables in the data analyses, data from eight climate variables were simultaneously obtained during this period. Significant climate variable identified by correlation analyses was executed to improve time series modeling as external repressors.

**Results:**

Among inpatients with HFMD, 248 (15.9%) were affected by EV71, 137 (8.8%) were affected by Pan-EV+, and 436 (28.0%) were affected by CA16. Optimal Univariate SARIMA model was identified: (2,0,3)(1,0,0)_52_ for inpatients, (0,1,0)(0,0,2)_52_ for outpatients as well as three serotypes (EV71, (1,0,1)(0,0,1)_52_; CA16, (1,0,1)(0,0,0)_52_; Pan-EV, (1,0,1)(0,0,0)_52_). Using climate as our independent variable, precipitation (PP) was first identified to be associated with inpatients (r = 0.211, *P* = 0.001), CA16-serotype (r = 0.171, *P* = 0.007) and outpatients (r = 0.214, *P* = 0.01) in partial correlation analyses, and was then shown a significant lag in cross-autocorrelation analyses. However, inclusion of PP [lag -3 week] as external repressor showed a moderate impact on the predictive performance of the SARIMA model described here-in.

**Conclusion:**

Climate patterns and HFMD incidences have been shown to be strongly correlated. The SARIMA model developed here can be a helpful tool in developing an early warning system for HFMD.

## Introduction

Hand foot and mouth disease (HFMD) represents a common viral infection that affects children of ages 5 years and younger [1, 2]. Originally identified more than five decades ago, HFMD presents with fever, sores on the hands, feet, mouth and buttocks [3]. HFMD has several causative agents, such as Enterovirus 71 (EV71) and Coxsackie virus A16 (CA16). Additionally, other strains have been reported to cause the illness [4–6]. Given the plethora of causative agents, the development of an effective vaccine for the prevention of HFMD has been hampered [7, 8].

Numerous outbreaks of HFMD were recently reported in mainland China [1]. The number of cases reported had increased yearly and has been well documented by the People's Republic of China Ministry of Health and Family Planning [9]. HFMD has a predictable outbreak incidence occurring every 2–3 years in affected countries [10]. However, given the complexity of the factors influencing HFMD outbreaks like the lack of resources to daunt the rate of infection, continued viral mutations, and climate changes favoring transmission of the disease, development of an early warning system was compelled.

Efforts are underway to identify the variables that expedite the HFMD epidemic [11–17]. Importantly, environmental determinants associated with HFMD outbreaks would provide an early warning system to identify potential outbreaks [18–20]. Multiple reports have suggested that climate variables may be used in developing a tool that will help forecast future outbreaks [21–24]. Huang and colleagues showed that in patients age 0–14 years the number of weekly reported cases of HFMD increased by 1.86% for every 1℃ increase. Additionally, for every 1% increase in relative humidity, there was a 1.42% increase in incidence [25]. The evidence provided by Huang and colleagues and others suggests that the use of statistical models in the development of an early warning system, such as those developed for other virally transmitted diseases, may be applied to the HFMD [25–29].

In this study, we undertook the task of developing a model to identify weather patterns associated with an increased incidence of HFMD from 2009 to late 2013 in Guangzhou city, one of the major trade cities in southern China. Feng and colleagues successfully applied Seasonal Autoregressive Integrated Moving Average (SARIMA) model to identify that weather variable (e.g. Temperature) was associated with incidence of HFMD [24]. Here we show that temperature, humidity and other meteorological factors highly correlate with the incidence of HFMD as indicated by the SARIMA model.

## Materials and Methods

### Ethics Statement

This study was approved by Zhujiang Hospital of Southern Medical University. It was also approved by the Ethics Committee of Zhujiang hospital. Written informed consent was obtained from the parents of every child participant enrolled in this study.

### Study Area

Guangzhou, the capital city of Guangdong Province and the third biggest city in China, is located in the Southern part of China and situated in the north hemisphere from 112° 57' to 114° 03' E longitude and 22° 26' to 23° 56' N latitude. The total area under the city's administration is 7,434.4 square kilometers (2,870.4 sq. mi). The total population of the city amounted to12.78 million by the end of 2012. Located just south of the Tropic of Cancer, Guangzhou has a humid subtropical weather influenced by the East Asian monsoon. The annual mean temperature ranges from 18°C to 25°C. The average annual rainfall is between 1,500mm and 2,000mm, with an average relative humidity of 77% [30].

### Meteorological Data

We obtained data for the meteorological variables at daily intervals from the National Meteorological Information Center (http://cdc.cma.gov.cn/). T, Average temperature (℃); TM, Maximum temperature (℃); Tm, Minimum temperature (℃); H, Humidity (%); VV, Visibility (Km); V, Mean wind speed (Km/h); VM, Maximum sustained wind speed (Km/h); PP, Precipitation amount (mm) were collected from a meteorological station in Guangzhou city. Daily diurnal variation in temperature was calculated by subtracting the maximum and minimum temperature. These data were available for the period from January 2009 to October 2013 without any missing values.

### Diagnosis criteria and specimen collection

The patients were identified with HFMD according to the diagnostic criteria defined by Chinese Ministry of Health and hospitalized only when meeting the criteria previously established[31,32]. Zhujiang Hospital is a HFMD-sentinel hospital in Guangzhou city and serves the surrounding areas. Participation in this study was voluntary and was proposed to all eligible patients until the target sample number was reached. Samples not taken or refusal of participation rate was approximately 9%. Viral serotypes (EV71, Pan-EV, and CA16) in 11,004 outpatients and 1,556 inpatients recruited from January 2009 to October 2013 were assessed by real time reverse-transcription polymerase chain reaction (RT-PCR) using stool samples.

### Laboratory Testing for Enterovirus

Stool specimens were collected from hospitalized HFMD patients enrolled in this study for use in RT-PCR. These samples were transported immediately at 4℃ to the clinical laboratory. A commercial licensed kit (Da An Gene Co. Ltd, lot no: CA16 YZB-0354-2009, EV-A71 YZB-0356-2009, Pan-Enterovirus: YZB-0355-2009) was recommended by the Center of Disease Control of China for detection of CV-A16, EV-A71, and Pan-Enterovirus. The detection method is based on one-step RT-PCR assay. The detection sensitivity of the kit is 1 × 10^3^ p.f.u/ml. A sample was considered positive for viruses if reaction growth curves crossed the threshold line within 35 cycles [12].

### Statistical Analyses

In this study, the SARIMA model was applied to predict the number of HFMD hospitalizations and the major enterovirus infections among Guangzhou City China and its surrounding areas. In general, SARIMA is one of several models used in forecasting time series data, in which three important terms are included: autoregressive (AR) term, data of present and past time points to be included in the model; differencing, transforming time series from non-stationary to stationary; moving average (MA) term, errors of present and past time points to be included in the model. Therefore, a SARIMA model was determined as (p, d, q) (P, D, Q) [s], where p, d, q were non-negative integers and indicated orders of non-seasonal AR terms, non-seasonal differencing and non-seasonal MA, respectively; P, D, Q were also non-negative integers and indicated orders of seasonal SAR terms, seasonal differencing and seasonal SMA terms, respectively; s indicated seasonal period (s = 52 weeks in this study). In order to fit the SARIMA model, the number of patients diagnosed with HFMD was first counted among seven consecutive days for inpatients (hospitalized) and outpatients (non-admitted). Inpatients infected with EV71, CA16, or Pan-EV were analyzed separately. R package “***forecast***” [33] was used to generate an optimal SARIMA model for each of the time series followed by four steps. First, square root transformation was performed to stabilize the variance of time series, and the kappa test [34] was used to test stationary status of the time series. Second, an optimal SARIMA model was obtained using the function “***auto*.*arima***”, model parameters (e.g., p, d, q, P, D, Q) were validated by autocorrelation function (ACF) plot and partial autocorrelation function (PACF) plot. Alternative SARIMA models were established by slightly changing model parameters. Third, the Akaike information criterion (AIC) and R squared (*R* ^2^) were also conducted to compare the goodness-of-fit among SARIMA models. A model with the lowest AIC and the highest *R* ^2^ values was considered to be optimal. Fourth, residuals of an optimal model were tested by the Box-Ljung test [35] to see if it was time-independent. For both inpatients and outpatients, the models developed by dividing the data file into two data sets: the data from 2009 to 2012 (estimation period) were used to construct a SARIMA model and those between 2012 and 2013(evaluation period) were used to validate the model.

We evaluated whether optimal SARIMA models incorporating weather variables have greater predictive power. To facilitate the selection of weather variables as external repressors, the Spearman rank correlation was first used to examine association between numbers of HFMD cases and meteorological parameters. To overcome the autocorrelation within each individual series, weather variables were then computed by pre-whitening using R packages **TSA**, and cross-autocorrelation analysis was used to assess associations between HFMD cases and each of weather variables over a range of time lags (a time lag was defined as the time span between climatic observation and the incidence of HFMD). Using the SARIMA model, the trend and seasonal components of the weather variable data were removed, for each weather variable multiple lag points were tested (-20 lag to +20 lag). Lagged weather variables that significantly associated with the number of HFMD cases were tested as external predictors in multivariate SARIMA model. The comparisons of the SARIMA with and without climatic variables were conducted. The predictive validity of the models was evaluated by calculating the root mean square error (RMSE), which measures the amount by which the fitted values differ from the observed values. The smaller the RMSE, the better the model is for forecasting. All above statistical analyses were carried out by R package version 3.1.0 and statistically significant was considered as *P* value <0.05.

## Results

### Patient surveillance data

**Fig 1:**
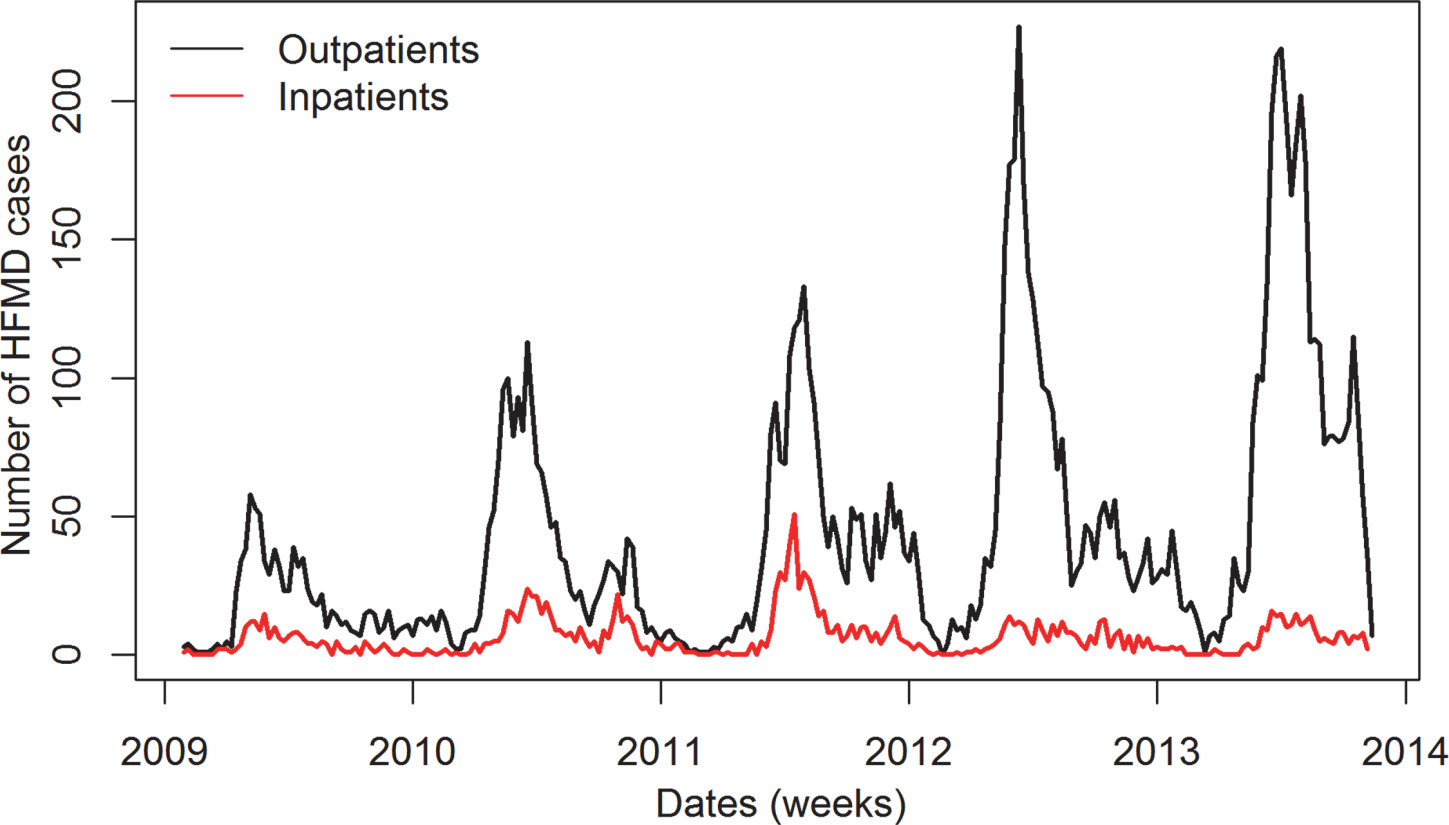
The number of HFMD cases collected from 2009–2013.

In this study, data was collected for inpatient and outpatient participants from January 2009 to October 2013. Within our study group, 32 of 1,588 inpatients and 118 of 11,122 outpatients were excluded for failing to meet inclusion criteria with respect to the definition of HFMD. The inpatient group consisted of 1,556 cases which provided stool samples for RT-PCR to assess viral serotype. Of these inpatients, 1,004 (64.5%) were males and the age ranged from one month to 14 years old with 94.9% ≤5 years old. Additionally, there were 162 severe cases and 11 deaths. Of all cases, the serotype detected was as follows: 248 (15.9%) EV71+, 436 (28.0%) CA16+, and 137 (8.8%) Pan-EV+ (Non- EV71/-CA16+). The outpatient group consisted of 11,004 participants. Of 11,004 outpatients, 6,540 (59.4%) were males and the age ranged from one month to 16 years old with 90.4% ≤5 years old (**Table 1**). As shown in **Fig. 1**, there were more outpatients than inpatients in each year throughout the study. Interestingly, the number of cases reported displayed a bimodal incidence rate. These peaks reached a maximum during April to July and again spiked in September to October.

**Table 1:** Descriptive statistics for HFMD cases collected from January 2009-October 2013.

**Variables**	**Total**	**Age**	**Gender**	**Severe cases**	**Deaths**
		**Range**	**Male Female**		
Case(in-patient)	1,556	One month-14 years old Under 5 years old 94.9%	1,004 552	162	11
Case(out-patient)	11,004	One month-16 years old Under 5 years old 90.4%	6,540 4,464	-	-

### Univariate ARIMA model

**Fig 2:**
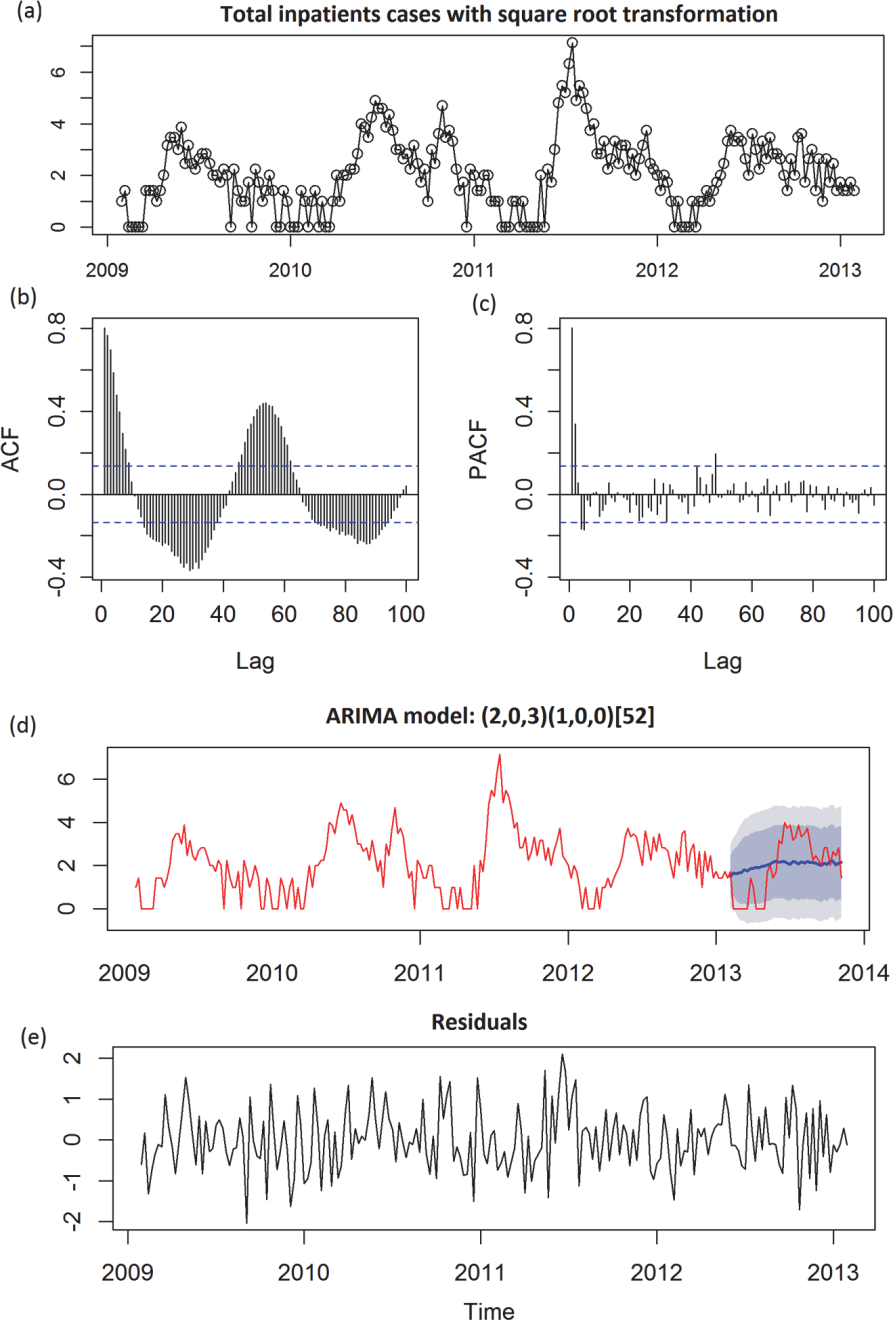
Univariate ARIMA analyses for all in-patients affected with HFMD. (a) Time series plot of total inpatients using raw data after square root transformation; (b) Autocorrelation (ACF) plot of total inpatients using raw data after square root transformation; (c) Partial ACF (PACF) plot of total inpatients using raw data after square root transformation; (d) Prediction plot after applying a SARIMA (2, 0, 3) (q, 0, 0)_52_ model; (e) Time series plot of residuals after applying a SARIMA (2, 0, 3) (q, 0, 0)_52_ model, shadow indicated 68% and 95% confidential interval. In ACF plot and PACF plot, x-axis gives the number of lags in weeks and the y-axis, the Dotted lines, indicate 95% confidence interval.

**Fig 3:**
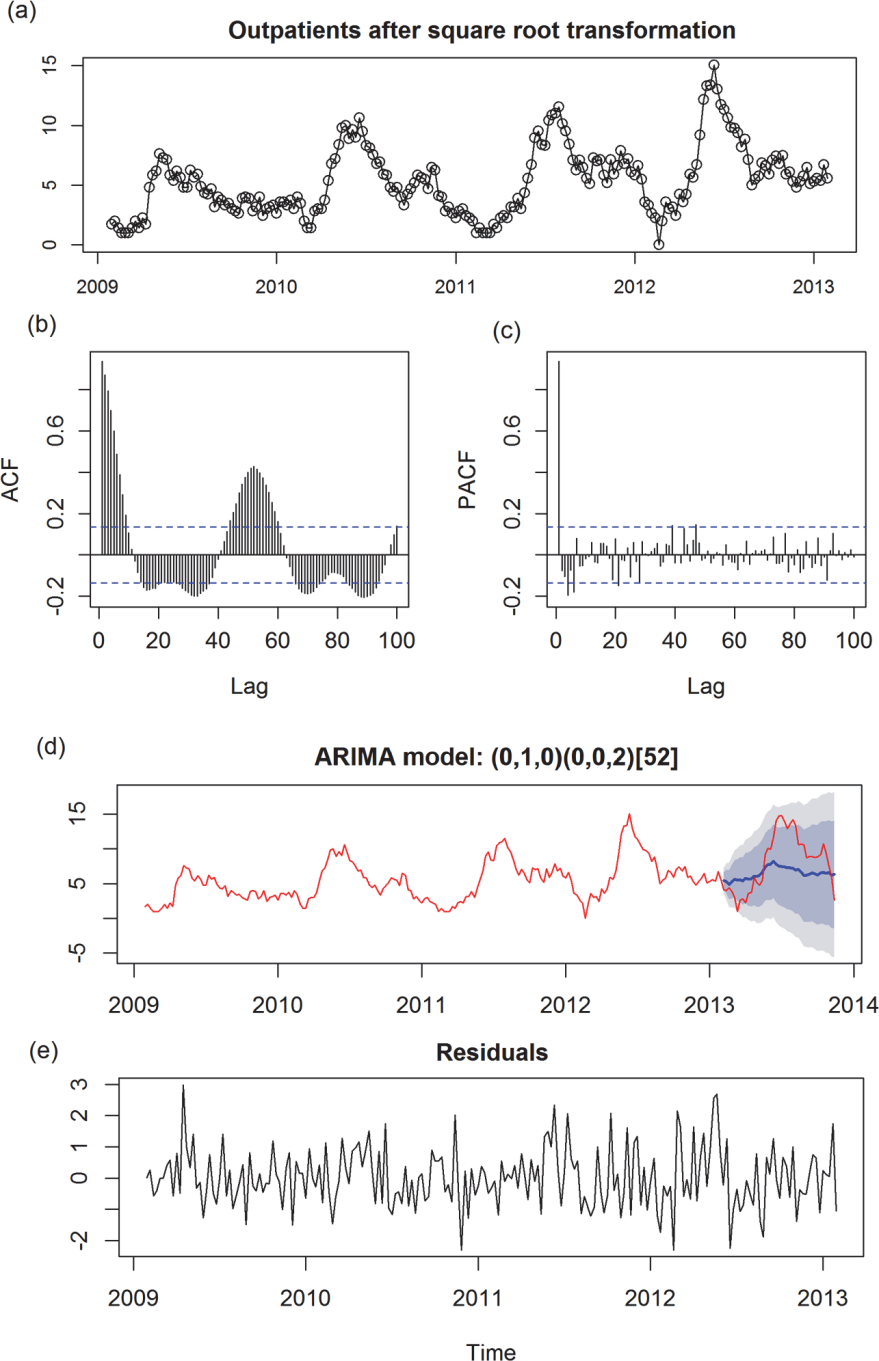
Univariate ARIMA analyses for all out-patients affected with HFMD. (a) Time series plot of total outpatients using raw data after square root transformation; (b) Autocorrelation (ACF) plot of total outpatients using raw data after square root transformation; (c) Partial ACF (PACF) plot of total outpatients using raw data after square root transformation; (d) Prediction plot after applying a SARIMA (0, 1, 0) (0, 0, 2)_52_ model; (e) Time series plot of residuals after applying a SARIMA (0, 1, 0) (0, 0, 2)_52_ model, shadow indicated 68% and 95% confidential interval. In ACF plot and PACF plot, x-axis gives the number of lags in weeks and the y-axis, the Dotted lines, indicate 95% confidence interval.

Before using SARIMA model, a square root transformation was performed to stabilize the variance of the time series. The plots of auto correlation function (ACF) and partial auto correlation function (PACF) showed the temporal dependence of the number of cases hospitalized with HFMD and confirmed the need to use a SARIMA model with seasonal (P, D, Q) and non-seasonal (p, d, q) parameters. For total inpatients, the time series plot was shown in **Fig. 2A**. Upon checking ACF and PACF (**Fig. 2B-2C**), p and q should be 2 and >5, kappa test indicated d = 0. We applied auto.arima function to detect a best model: (2,0,3)(1,0,0)_52_ with the lowest AIC (AIC = 491.98) and the highest *R* ^2^ values (*R* ^2^ = 0.7080), and the prediction was shown as **Fig. 2D**. To assess the fitness of the model, residuals were applied and the results showed an independent pattern (*P*_Box-Ljung_ = 0.8777, **Fig. 2E**). For total outpatients, the time series plot was shown after transformation **Fig. 3A**. Upon checking ACF and PACF (**Fig. 3B-3C**), p and q should be 1 and >5, kappa test indicated d = 1. After 1^st^ order of differencing, we applied auto.arima function to detect a best model: (0,1,0)(0,0,2)_52_ with lowest AIC (AIC = 576.15) and highest *R* ^2^ values (*R* ^2^ = 0.8925), and prediction was shown as **Fig. 3D**. To detect whether it is a good model, residuals after apply above model showed an independent pattern (*P*_Box-Ljung_ = 0.0669, **Fig. 3E**). Additionally, we also analyzed SARIMA models for subgroups of inpatients. For inpatients with EV71, the best model was (1,0,1)(0,0,1)_52_ (**S1 Fig.**); for inpatients with CA16, the best model was (1,0,1)(0,0,0)_52_ (**S2 Fig.**). For inpatients with EV, the best model was (1, 0, 0) (0, 0, 0)_52_ (**S3 Fig.**). Detailed information on these models are presented in **Table 2**.

**Table 2:** Best predictive SARIMA models for inpatients and Outpatients.

**Items**	**Inpatients total**	**Inpatients with EV71**	**Inpatients with CA16**	**Inpatients with Pan-EV**	**Outpatients total**
SARIMA Models	(2,0,3)(1,0,0)_52_	(1,0,1)(0,0,1)_52_	(1,0,1)(0,0,0)_52_	(1,0,1)(0,0,0)_52_	(0,1,0)(0,0,2)_52_
AIC	491.98	386.26	458.16	314.62	576.15
*R* ^2^	0.7080	0.5290	0.4830	0.4418	0.8925
RMSE training	0.7522	0.5917	0.7091	0.5029	0.9437
RMSE validating	1.2221	0.4513	0.9208	0.4304	3.7297
*P*_Box-Ljung_	0.8777	0.9175	0.9631	0.9989	0.0669
AR1	0.9300	0.9086	0.8676	0.9361	-
AR2	-0.0793	-	-	-	-
MA1	-0.4542	-0.5021	-0.3930	-0.6310	-
MA2	0.2315	-	-	-	-
MA3	0.1476	-	-	-	-
SAR1	0.0928	0.1778	-	-	-
SMA1	-	-	-	-	0.2134
SMA2	-	-	-	-	0.0941

SARIMA: Seasonal Autoregressive Integrated Moving Average; autoregressive, MA: moving average, SAR: seasonal autoregressive.

SMA, seasonal moving average; AIC, Akaike information criterion; PBox-Ljung, Ljung-Box test, RMSE: Root Mean Square;

### Partial Correlation analyses between HFMD cases and eight weather variables

Accounting for these inter-correlations, associations between meteorological factors and the number of HFMD hospitalization were then analyzed using partial correlations: detection of any of the pathogens was associated with average atmospheric temperatures. As shown in **Table 3**, total inpatients were statistically associated with PP (r = 0.211, *P* = 0.001). Inpatients with EV71 were significantly correlate with T (r = -0.179, *P* = 0.005) and Tm (r = 0.271, *P*<0.001). Inpatients with EV were significantly correlate with PP (r = 0.171, *P* = 0.007). Total outpatients were significantly correlate with T (r = 0.165, *P* = 0.009), Tm (r = -0.216, *P* = 0.001), H(r = 0.198, *P* = 0.002) and PP(r = 0.214, *P* = 0.001).

**Table 3:** Partial correlation between HFMD and nine climate variables.

**Variables**	**Inpatients total**		**Inpatients with EV71**		**Inpatients with CA16**		**Inpatients with Pan-EV**		**Outpatients total**	
	**r**	***P***	**R**	***P***	**r**	***P***	**r**	***P***	**r**	***P***
T	0.061	0.342	-**0.179**	**0.005**	0.085	0.187	-**0.234**	**0.000**	**0.165**	**0.009**
TM	-0.040	0.537	0.093	0.146	-0.040	0.539	**0.128**	**0.045**	-0.038	0.560
Tm	0.002	0.972	**0.271**	**0.000**	-0.079	0.222	**0.325**	**0.000**	-**0.216**	**0.001**
H	-0.080	0.216	-0.109	0.089	-0.057	0.379	-0.117	0.068	**0.198**	**0.002**
VV	-0.075	0.244	0.051	0.425	-0.084	0.193	0.089	0.167	-0.121	0.059
V	0.003	0.968	0.025	0.699	-0.027	0.676	-0.031	0.632	0.047	0.461
VM	0.037	0.565	-0.034	0.599	0.058	0.371	0.020	0.757	0.066	0.308
PP	**0.211**	**0.001**	0.058	0.370	**0.171**	**0.007**	0.024	0.709	**0.214**	**0.001**

T, Temperature (°C); TM, Maximum temperature (℃); Tm, Minimum temperature (℃); H, Humidity (%); VV, Visibility (Km); V, Mean wind speed (Km/h); VM, Maximum sustained wind speed (Km/h); PP, Precipitation amount (mm). r, correlation coefficient; *P*, p value obtained from Partial correlation analyses.

### Multivariate SARIMA model integrating weather variables

Next, we asked whether most HFMD-associated weather variables could help refine the prediction models. To include climatic variables (time series) as external variables, a multivariate SARIMA model was applied to the time series. We first removed the trend and seasonal components of each time series through SARIMA modeling. To further validate the results of partial correlation analyses, we then applied cross-autocorrelation analyses to compute the lag of weather variable that was significantly associated with HFMD cases, implemented by R packages **TSA**. In order to adjust autocorrelations of each of weather variables, weather variables were pre-whitening before analyses. CCF plots were displayed as **S4 Fig.**, and the results of cross-autocorrelation analyses were summered in **Table 4.** For inpatients, the most associated weather variables were H lag-1 week and PP lag -3 week. For inpatient with EV71, the most associated weather variables were H lag-2 week and PP lag -3 week. For inpatient with CA16, the most associated weather variables were PP lag 1 week. For inpatient with EV, the most associated weather variables were H lag-1 week and PP lag -6 week. For outpatients, the most associated weather variables were Tm lag -1 week, H lag-2 week and PP lag -3 week.

**Table 4:** Cross-autocorrelation of nine climate variables with HFMD cases.

**Groups**	**climate variable**	**Lags (week)**	**r**	***P***
Inpatients total	H[–1]	-1	0.161	<0.05
	PP[–3]	-3	0.259	<0.05
Inpatients with EV71	H[–2]	-2	0.166	<0.05
	PP[–3]	-3	0.265	<0.05
Inpatients with CA16	PP[1]	1	0.215	<0.05
Inpatients with Pan-EV	H[–1]	-1	0.150	<0.05
	PP[–6]	-6	0.235	<0.05
Outpatients total	Tm[–1]	-1	0.165	<0.05
	H[–1]	-1	0.203	<0.05
	PP[–3]	-3	0.254	<0.05

Tm, Minimum temperature (℃); H, Humidity (%); PP, Precipitation amount (mm).

r, correlation; coefficient; *P*, p value obtained from cross-autocorrelation analyses after pre-whitening.

**Fig 4:**
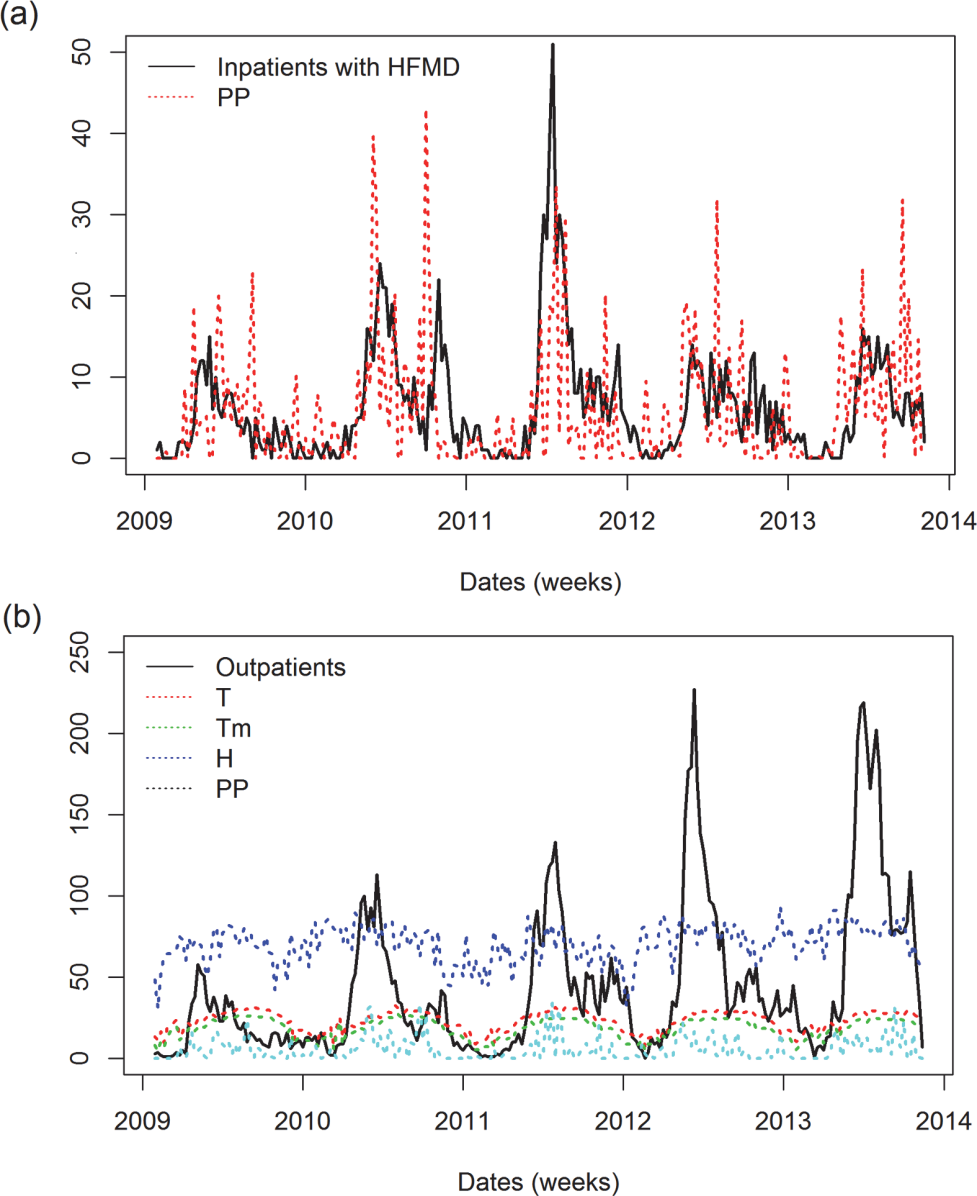
Data visualization of all in-patients and out-patients integrating with climate variables. (a) Time series plot of total inpatients after square root transformation and its most correlated climate variable PP (Precipitation amount, mm). (b) Time series plot of total outpatients after square root transformation and its most correlated climate variables: T (Temperature, °C), Tm (Minimum temperature, °C), H (Humidity, %) and PP (Precipitation amount, mm).

**Fig 5:**
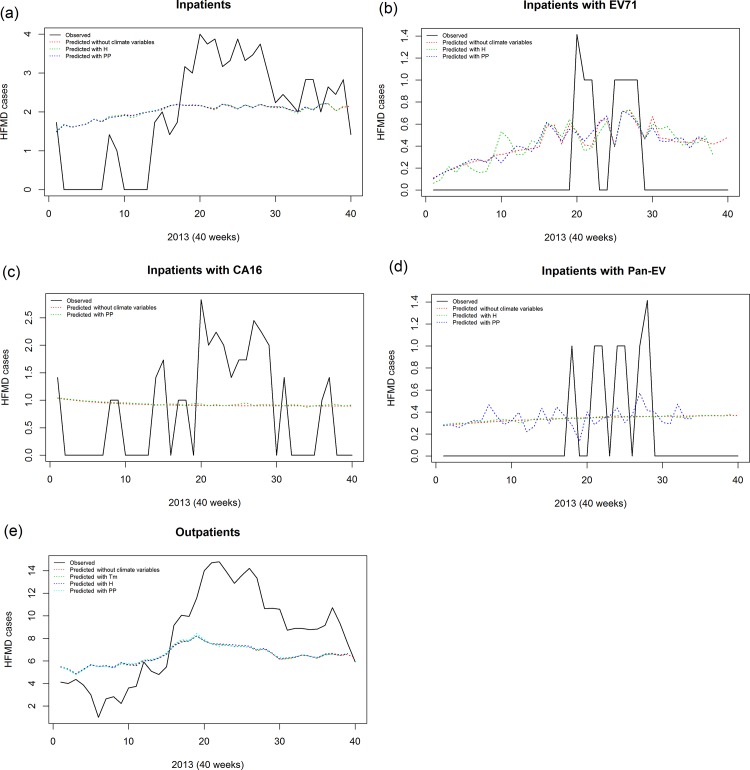
Prediction analyses of HFMD cases integrating with most associated climate variables. (a) Prediction curves of total inpatients using raw data, Univariate SARIMA model and multivariate SARIMA models integrating PP; (b) Prediction curves of total inpatients with EV71 using raw data, Univariate SARIMA model and multivariate SARIMA models integrating H and PP; (c) Prediction curves of total inpatients with CA16 using raw data, Univariate SARIMA model and multivariate SARIMA models integrating PP; (d) Prediction curves of total inpatients with EV using raw data, Univariate SARIMA model and multivariate SARIMA models integrating H and PP; (e) Prediction curves of total inpatients using raw data, Univariate SARIMA model and multivariate SARIMA models integrating Tm, H and PP.

Second, the identification of weather variables that significantly correlated with HFMD hospitalizations were tested with univariate SARIMA models, which were carried out by including external independent variables. In order to visualize the correlation between HFMD cases and weather variables, we plotted inpatients together with most associated weather variable PP (**Fig. 4A**) and outpatients together with most associated weather variables T, Tm, H, PP (**Fig. 4B**), suggesting weather variable could be highly correlated with HFMD cases. We first removed the trend and seasonal parts of each time series, and included the remaining irregular part as an external repressor in the SARIMA model; the previously established models were then modified accordingly. As shown in **Table 5**, incorporating weather variables could have different impacts on the ARIMA model. For total inpatients, H lag -1 week and PP lag -3 week increased on AIC, but *R* ^2^ and RMSE did not change significantly. For total outpatients, PP lag -3 week can decrease AIC but increased *R* ^2^ and RMSE. Overall, **Fig. 5** shows that our SARIMA models have a good prediction on HFMD cases as well as subgroups and incorporating most correlated weather variables did not substantially improve prediction, suggesting a stochastic mechanism of interactions between weather variables and HFMD cases.

**Table 5:** Predictive models integrating best associated climate variables.

**Items**	**Climate variables [Lags]**	**AIC**	***R*^2^**	**RMSE training**	**RMSE validating**	***P*_Box-Ljung_**	**AR1**	**AR2**	**MA1**	**MA2**	**MA3**	**SAR1**	**SMA1**	**SMA2**	**Climate**
Inpatients total	H[–1]	493.82	0.7082	0.7519	1.2291	0.8770	0.9216	-0.0723	-0.4460	0.2284	0.1520	0.0931	-	-	-0.0024
	PP[–3]	493.93	0.7081	0.7521	1.2556	0.8928	0.9460	-0.0927	-0.4671	0.2349	0.1414	0.0933	-	-	-0.0019
Inpatients with EV71	H[–2]	385.19	0.5364	0.5872	0.4607	0.8810	0.9050	-	-0.4844				0.1809	-	0.0088
	PP[–3]	387.18	0.5316	0.5902	0.4447	0.9070	0.9057	-	-0.4889	-	-	-	0.1784	-	-0.0068
Inpatients with CA16	PP[1]	458.02	0.4836	0.7091	0.9211	0.9333	0.8651	-	-0.3881	-	-	-	-	-	-0.0024
Inpatients with Pan-EV	H[–1]	316.55	0.4420	0.5028	0.4311	0.9988	0.9361	-	-0.6305	-	-	-	-	-	-0.0011
	PP[–6]	311.25	0.4560	0.4965	0.4417	0.9973	0.9310	-	-0.6058	-	-	-	-	-	0.0135
Outpatients total	Tm[–1]	574.98	0.8928	0.9427	3.7715	0.0613	-	-	-	-	-	-	0.2168	0.0916	-0.0189
	H[–1]	574.80	0.8929	0.9422	3.7506	0.0500	-	-	-	-	-	-	0.2074	0.1047	-0.0054
	PP[–3]	573.91	0.8933	0.9400	3.8300	0.0600	-	-	-	-	-	-	0.2244	0.0912	0.0085

SARIMA: Seasonal Autoregressive Integrated Moving Average; autoregressive, MA: moving average, SAR: seasonal autoregressive.

SMA, seasonal moving average; AIC, Akaike information criterion; PBox-Ljung, Ljung-Box test, RMSE: Root Mean Square;

## Discussion

The incidence of HFMD has been previously documented in a myriad of cities of the Pacific Asian region [1, 36–38]. In this report, we undertook the task of identifying potential underlying factors influencing the recent outbreaks of Hand, Foot and Mouth Disease (HFMD) in Guangzhou, a major trading city in southern China from January 2009 to October 2013. Of 12,560 enrolled subjects, 11,004(87.6%) were outpatient and 1,556 (12.4%) were inpatient. Within the inpatient population, the male to female ratio was1.46:1 while 94.9% of them were under five years. Of the inpatient cases, 162 were considered severe, requiring ICU monitoring, and 11 capitulated (**Table 1**). Our findings are in agreement with published reports and further confirm that the yearly increased incidence of HFMD is bimodal and typically occurs during April-July and September-October (**Fig. 1**) [36–38] suggesting that preliminary preparations can be put in place at HFMD-sentinel facilities to prevent outbreaks during specific seasons of the year.

Less than optimal personal hygiene and contact with an infected individual in addition to weather changes have been identified as critical factors for the increased incidence of HFMD [12]. To determine whether the observed peak incidence of HFMD (Fig. 1) was associated with changes in weather patterns, we employed the SARIMA modeling. This tool has been successfully used to interpret surveillance data, as well as to incorporate external factors such as weather variables which increase its predictive power[27, 39]. The model showed that an increase in average temperature (T) influenced the number of outpatient diagnoses (**Table 3 and Fig. 4A**) (rather than inpatients) and had a strong correlation with the number of EV71^+^ (**Table 3 and S5A Fig.**) and Pan-EV^+^ inpatients (**Table 3 and S5C Fig.**)except CA16^+^ inpatients (Table 3). Importantly, these results were acquired using the univariate model. However, when the weather variables were integrated into a multivariate model, the results showed that (T) failed to maintain its predictive power, while H and PP maintained their predictive power (Table 4). Inversely, an increase in the maximum temperature (TM) was only correlated with increased inpatient Pan-EV^+^ cases (**Table 3 and S5C Fig.**). An increase in the minimum temperature (Tm), however, showed a similar effect as the change in the TM (**Table 3 and S5C Fig.**). Others have shown that an increase in temperature correlates with an overall increase in consultation rate [25]. Our data, and that of others [25], suggests that while temperature has a strong correlation with increased incidence of HFMD, it has little to no detectable impact on the severity of the cases during an outbreak. Of note, there were inconsistencies between partial correlation analyses and cross-autocorrelation. For example, temperature (T) was significantly correlated with inpatients with EV71^+^ and Pan-EV^+^ (Table 3), but none of lag of T was found to be independently correlated with HFMD cases when controlling autocorrelation within time series, suggesting stochastic mechanism existed between T and HFMD cases and latent confounding factor [40].

Similar to temperature, an increase in humidity (H) showed a strong correlation with the total number of outpatient cases (**Table 3 and Fig. 4B**) while remaining uninfluential on the inpatient population when using the univariate model (**Table 3**). Furthermore, an increase in H showed no effect on the serotypes detected in the patient subpopulations. Like temperature, humidity failed to show a strong correlation in a multivariate model (Table 4). Huang et.al [25] showed that the incidence of HFMD in Guangzhou had high association with increased humidity. Their data were generated using a different model thus providing validity to our model which used different weather variable data, patient population, and in different years but still showed similar results. Our data further support those finding and suggest that humidity can be reliably used to predict an outbreak.

In addition, precipitation was also investigated; our data show that there was a strong correlation between increased precipitation and the increased number of outpatients and inpatients with HFMD as well as patients with CA16 serotypes (**Table 3, Table 4, Fig. 4A and 4B**). Furthermore, when analyzing sub-phenotypes of inpatients, the CA16 serotype was significantly correlated with increased precipitation but EV71 and Pan-EV were not (Table 3). While this is not the first report that investigates the impact of precipitation on HFMD [25], to our knowledge, we are the first to report that precipitation may have an impact on the serotypes detected in the inpatient population. These data suggest that if the severity of the illness depends on viral strain, if precipitation should increase during peak periods, facilities may use this information to better prepare for patient over flow.

While the current study focused on weather variables and their impact on the increased incidence of HFMD, there remain limitations to our findings. First, while we included eight weather variables, given the numerous variables available, we could have considered inclusion of more of them to further validate the use of the present model. Second, the inclusions of different geographic areas in other parts of the world are warranted in future analyses to validate the SARIMA model identified. Furthermore, we utilized data obtained from Zhujiang Hospital only. Admittedly, the population within this study comes from within Guangzhou and its surrounding areas and the frank number of Guangzhou residents is undeterminable. In addition, the restricted access to the surveillance data limits our study to Zhujiang Hospital and understand that a selection bias may exist which is beyond the scope of this manuscript. Lastly, we did not assess the relationship between age and sex and HFMD in our study population due to subsets from stratification that could decrease statistical power and lead to unreliable results.

SARIMA has been widely used to establish predictable models to describe the pattern of time series data [24]. In this study, we first established optimal univariate SARIMA models for HFMD outbreaks with AICs and the highest *R* ^2^. We then evaluated if weather variables (e.g. humidity and precipitation) significantly correlated with HFMD outbreaks, then developed multivariate SARIMA model by including weather variables as external repressors, which did not dramatically improve the SARIMA model and might be explained by two factors: (1) a latent interaction effect between weather and HFMD repressors (e.g. AR and MA terms) may exist; (2) suggests a stochastic mechanism of interactions between weather variables and HFMD cases [24]. The predictive power of a SARIMA model, cross-autocorrelation and partial correlation like the ones used in this report to determine the correlation between HFMD and weather variables heavily rely on a rationally designed study. Here the data for each weather variable was collected daily. In doing so, we minimized the probability of under or over interpreting out results due to missing data points that may occur when the data is collected weekly or at larger intervals. In addition, Zhujiang Hospital is a sentinel facility for HFMD in Guangzhou. As such, many patients come for consultation regarding symptoms of HFMD. By collecting all data points at one facility tight control of patient samples can be accomplished, patient diagnosis remains consistent, serotypes are identically identified, and only patients meeting criteria are included in the study. By minimizing the variability introduced by experimental design we anticipate that the present model may serve as an early warning system of future outbreaks of HFMD.

## Supporting Information

S1 Fig Univariate ARIMA analyses for all in-patients affected with EV71. (a) Time series plot of total inpatients with EV71 using raw data after square root transformation; (b) Autocorrelation (ACF) plot of total inpatients with EV71 using raw data after square root transformation; (c) Partial ACF (PACF) plot of total inpatients with EV71 using raw data after square root transformation; (d) Prediction plot after applying a SARIMA (1, 0, 1) (0, 0, 1)_52_ model; (e) Time series plot of residuals after applying a SARIMA (1, 0, 1) (0, 0, 1)_52_ model, shadow indicated 68% and 95% confidential interval. In ACF plot and PACF plot, x-axis gives the number of lags in weeks and the y-axis, the Dotted lines, indicate 95% confidence interval.
(DOCX)

S2 Fig Univariate ARIMA analyses for all in-patients affected with CA16. (a) Time series plot of total inpatients with CA16 using raw data after square root transformation; (b) Autocorrelation (ACF) plot of total inpatients with CA16 using raw data after square root transformation; (c) Partial ACF (PACF) plot of total inpatients with EV71 using raw data after square root transformation; (d) Prediction plot after applying a SARIMA (1, 0, 1) (0, 0, 0)_52_ model; (e) Time series plot of residuals after applying a SARIMA (1, 0, 1) (0, 0, 0)_52_ model, shadow indicated 68% and 95% confidential interval. In ACF plot and PACF plot, x-axis gives the number of lags in weeks and the y-axis, the Dotted lines, indicate 95% confidence interval.
(DOCX)

S3 Fig Univariate ARIMA analyses for all in-patients affected with Pan-EV. (a) Time series plot of total inpatients with EV using raw data after square root transformation; (b) Autocorrelation (ACF) plot of total inpatients with EV using raw data after square root transformation; (c) Partial ACF (PACF) plot of total inpatients with EV using raw data after square root transformation; (d) Prediction plot after applying a SARIMA (1, 0, 1) (0, 0, 0)52 model; (e) Time series plot of residuals after applying a SARIMA (1, 0, 1) (0, 0, 0)52 model, shadow indicated 68% and 95% confidential interval. In ACF plot and PACF plot, x-axis gives the number of lags in weeks and the y-axis, the Dotted lines, indicate 95% confidence interval.
(DOCX)

S4 Fig Cross-autocorrelation analyses of all in-patients and outpatients and eight climate variables. Cross-autocorrelation analyses were applied to total inpatients (a), inpatients with EV71 (b), inpatients with CA16(c), inpatients with Pan-EV (d), and outpatients (e). T, Temperature (°C); TM, Maximum temperature (°C); Tm, Minimum temperature (°C); H, Humidity (%); VV, Visibility (Km); V, Mean wind speed (Km/h); VM, Maximum sustained wind speed (Km/h); PP, Precipitation amount (mm). X-axis gives the number of lags in weeks and the y-axis, the Dotted lines, indicate 95% confidence interval.
(DOCX)

S5 Fig Data visualization of inpatients affected with EV71 (a), CA16 (b), Pan-EV (c) integrating with climate variables. T, Temperature (°C); TM, Maximum temperature (°C); Tm, Minimum temperature (°C); H, Humidity (%); VV, Visibility (Km); V, Mean wind speed (Km/h); VM, Maximum sustained wind speed (Km/h); PP, precipitation amount (mm).
(DOCX)
