# Supplementary material for: Time Series Analyses of Hand, Foot and Mouth Disease Integrating Weather Variables
Source: PLoS One. 2015 Mar 2;10(3):e0117296. doi: 10.1371/journal.pone.0117296 (PMC4346267; doi:10.1371/journal.pone.0117296)
Supplement: S3 Fig — (a) Time series plot of total inpatients with EV using raw data after square root transformation; (b) Autocorrelation (ACF) plot of total inpatients with EV using raw data after square root transformation; (c) Partial ACF (PACF) plot of total inpatients with EV using raw data after square root transformation; (d) Prediction plot after applying a SARIMA (1, 0, 1) (0, 0, 0)52 model; (e) Time series plot of residuals after applying a SARIMA (1, 0, 1) (0, 0, 0)52 model, shadow indicated 68% and 95% confidential interval. In ACF plot and PACF plot, x-axis gives the number of lags in weeks and the y-axis, the Dotted lines, indicate 95% confidence interval. (DOCX) [file pone.0117296.s003.docx]

**
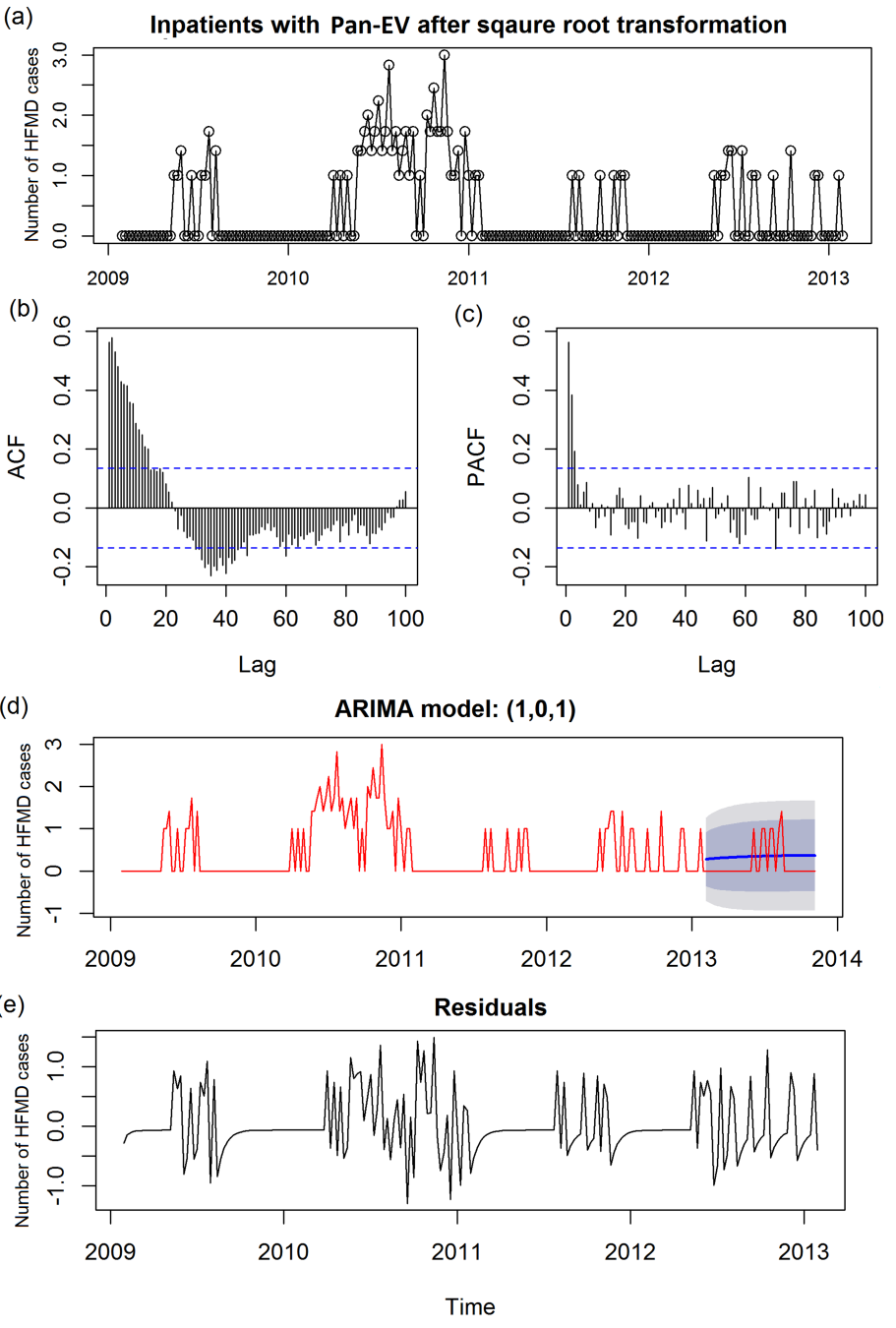
**

**Figure S3**. Univariate ARIMA analyses for all in-patients affected with Pan-EV

**Figure S5**. Data visualization of inpatients affected with EV71 (a), CA16 (b), Pan-EV (c) integrating with climate variables
