# Supplementary material for: Time Series Analyses of Hand, Foot and Mouth Disease Integrating Weather Variables
Source: PLoS One. 2015 Mar 2;10(3):e0117296. doi: 10.1371/journal.pone.0117296 (PMC4346267; doi:10.1371/journal.pone.0117296)
Supplement: S4 Fig — Cross-autocorrelation analyses were applied to total inpatients (a), inpatients with EV71 (b), inpatients with CA16(c), inpatients with Pan-EV (d), and outpatients (e). T, Temperature (°C); TM, Maximum temperature (°C); Tm, Minimum temperature (°C); H, Humidity (%); VV, Visibility (Km); V, Mean wind speed (Km/h); VM, Maximum sustained wind speed (Km/h); PP, Precipitation amount (mm). X-axis gives the number of lags in weeks and the y-axis, the Dotted lines, indicate 95% confidence interval. (DOCX) [file pone.0117296.s004.docx]

**
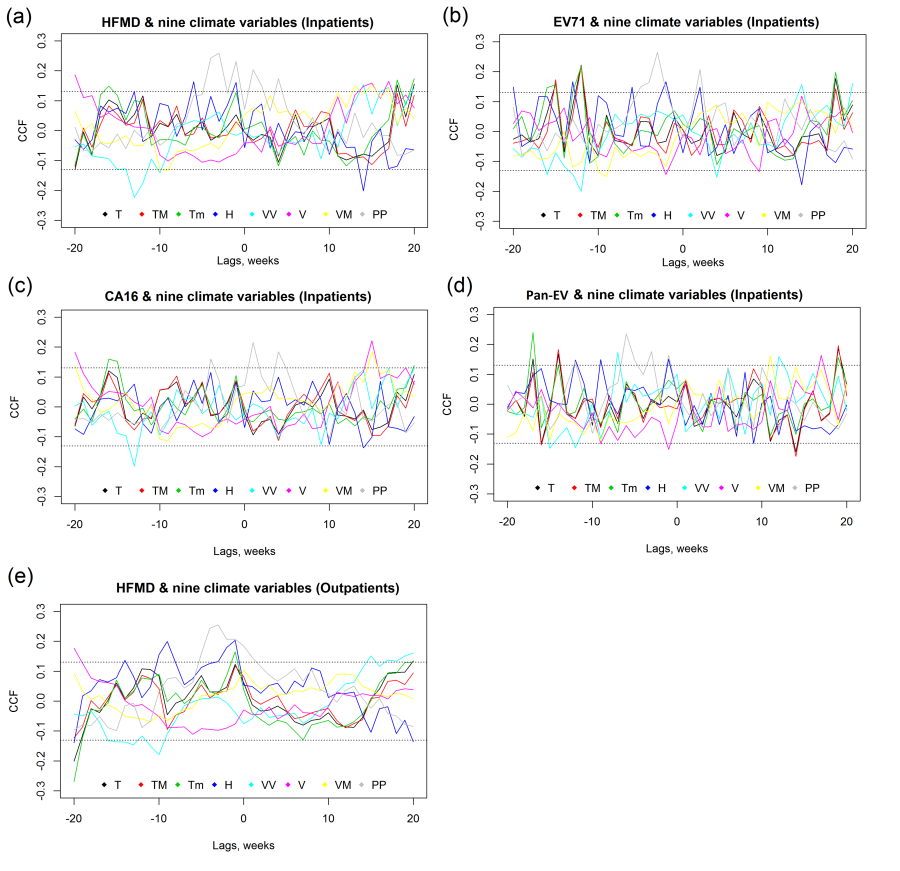
**

**Figure S4**. Cross-autocorrelation analyses of all in-patients and outpatients and eight climate variables
