# Supplementary material for: Time Series Analyses of Hand, Foot and Mouth Disease Integrating Weather Variables
Source: PLoS One. 2015 Mar 2;10(3):e0117296. doi: 10.1371/journal.pone.0117296 (PMC4346267; doi:10.1371/journal.pone.0117296)
Supplement: S5 Fig — T, Temperature (°C); TM, Maximum temperature (°C); Tm, Minimum temperature (°C); H, Humidity (%); VV, Visibility (Km); V, Mean wind speed (Km/h); VM, Maximum sustained wind speed (Km/h); PP, precipitation amount (mm). (DOCX) [file pone.0117296.s005.docx]

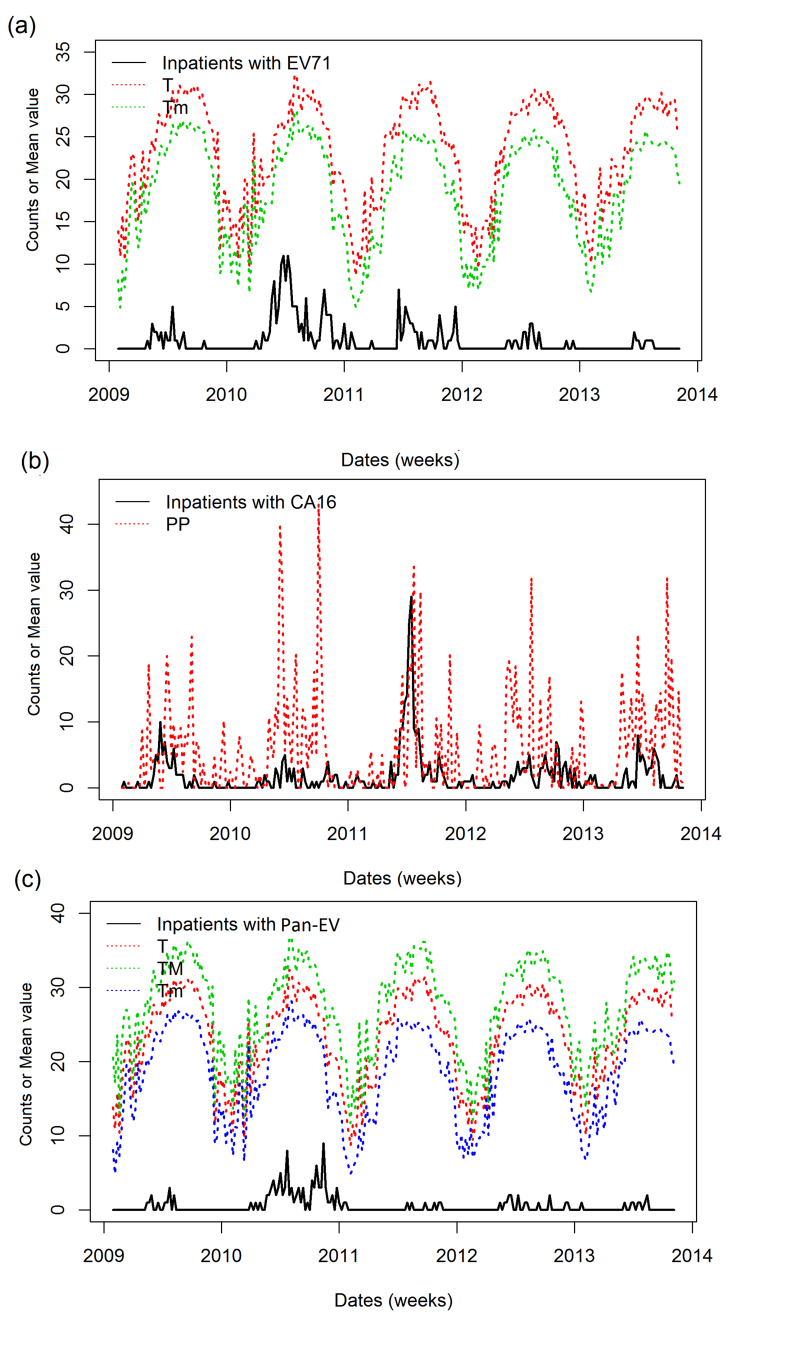


**Figure S5**. Data visualization of inpatients affected with EV71 (a), CA16 (b), Pan-EV (c) integrating with climate variables
